# Repression of FGF signaling is responsible for *Dnmt3b* inhibition and impaired *de novo* DNA methylation during early development of *in vitro* fertilized embryos

**DOI:** 10.7150/ijbs.51607

**Published:** 2020-10-03

**Authors:** Wei Fu, Yuan Yue, Kai Miao, Guangyin Xi, Chao Zhang, Wenjuan Wang, Lei An, Jianhui Tian

**Affiliations:** National Engineering Laboratory for Animal Breeding; Key Laboratory of Animal Genetics, Breeding and Reproduction of the Ministry of Agriculture; College of Animal Science and Technology, China Agricultural University, No.2 Yuanmingyuan West Road, Beijing 100193, P. R. China.

**Keywords:** *in vitro* fertilization, DNA methylation, *Dnmt3b*, FGF signaling

## Abstract

Well-orchestrated epigenetic modifications during early development are essential for embryonic survival and postnatal growth. Erroneous epigenetic modifications due to environmental perturbations such as manipulation and culture of embryos during *in vitro* fertilization (IVF) are linked to various short- or long-term consequences. Among these, DNA methylation defects are of great concern. Despite the critical role of DNA methylation in determining embryonic development potential, the mechanisms underlying IVF-associated DNA methylation defects, however, remains largely elusive. We reported herein that repression of fibroblast growth factor (FGF) signaling as the main reason for IVF-associated DNA methylation defects. Comparative methylome analysis by postimplantation stage suggested that IVF mouse embryos undergo impaired *de novo* DNA methylation during implantation stage. Further analyses indicated that *Dnmt3b*, the main *de novo* DNA methyltransferase, was consistently inhibited during the transition from the blastocyst to postimplantation stage (Embryonic day 7.5, E7.5). Using blastocysts and embryonic stem cells (ESCs) as the model, we showed repression of FGF signaling is responsible for *Dnmt3b* inhibition and global hypomethylation during early development, and MEK/ERK-SP1 pathway plays an essential mediating role in FGF signaling-induced transcriptional activation of *Dnmt3b.* Supplementation of FGF2, which was exclusively produced in the maternal oviduct, into embryo culture medium significantly rescued *Dnmt3b* inhibition. Our study, using mouse embryos as the model, not only identifies FGF signaling as the main target for correcting IVF-associated epigenetic errors, but also highlights the importance of oviductal paracrine factors in supporting early embryonic development and improving *in vitro* culture system.

## Introduction

IVF is one of the most effective and successful assisted reproductive technologies for treating infertility that affects ~15% of couples globally. Currently, it is estimated that more than 9 million babies have been born worldwide since the first IVF baby was born in 1978, and IVF contributes to 1-5% of all newborns in developed countries 1. Although the great majority of IVF-conceived offspring are in good health, increasing epidemiologic analyses in humans and laboratory studies in animals show that IVF is associated with various short- or long-term consequences, including pregnancy complications, preterm birth, low birth weight, birth defects 2-6, as well as higher disease risks in later life, such as heart disease, diabetes, and hypertension 7-11.

Mammalian early development is controlled by a series of spatiotemporally regulated epigenetic modifications that are critical for acquiring developmental potential. In particular, from preimplantation to postimplantation stage, environmental perturbations such as manipulation and culture of embryos *in vitro*, can affect the epigenetic programming of the developing embryos, and thus leading to erroneous epigenetic modifications, which are expected to be linked to those various short- or long-term consequences in IVF embryos and offspring 11-13. Among the IVF-associated epigenetic errors, DNA methylation defects are extensively studied. Earlier studies that reported imprinting disorders in IVF-conceived Angelman (AS) and Beckwith-Wiedemann syndromes (BWS) offspring 14-16, have raised safety concerns of clinical use of IVF. After that, imprinting loss of specific loci, as well as globally altered methylome, have been reported repeatedly in IVF-conceived embryos and offspring in humans 17, 18, domestic animals 19-21, and mouse models 22-25. Despite the critical role of DNA methylation in determining embryonic development and postnatal growth, the mechanisms underlying IVF-associated DNA methylation defects, however, remains largely elusive. Thus, there has never been potential strategy for preventing or correcting those disturbances.

Using methylated DNA immunoprecipitation-sequencing (MeDIP-seq) data that profiles DNA methylation maps from *in vivo* (IVO) and IVF mouse postimplantation embryos, which have established static DNA methylation patterns following* de novo* DNA methylation 26, 27, we found IVF processes induced global hypomethylation in postimplantation embryos. This finding suggests an impaired *de novo* DNA methylation, the critical epigenetic event that is prerequisite for re-establishing the DNA methylation patterns from a globally hypomethylated state in blastocysts to relatively static DNA methylation patterns in postimplantation embryos. Given the DNMT3B is the main enzyme required for *de novo* DNA methylation during early implantation 26, 28, we have explored the mechanism underlying impaired *de novo* DNA methylation by focusing on the expression patterns of *Dnmt3b* in IVF embryos, as well as the upstream signaling responsible for transcriptional regulation of *Dnmt3b*. Our study identifies repression of FGF signaling as one of the main barriers that induces DNA methylation defects in IVF postimplantation embryos, and provides a promising approach for preventing or correcting IVF-associated epigenetic errors.

## Materials and Methods

### Mouse embryo preparation and collection

All experimental procedures were approved by and performed in accordance with the guidelines of the Institutional Animal Care and Use Committee of China Agricultural University. Crlj:CD1 (ICR) mice used in present study were provided by SPF Biotechnology Co., Ltd (Beijing, China). Female mice were superovulated by an i.p. injection of 5 IU pregnant mare serum gonadotrophin (PMSG, Ningbo Hormone Product Co., Ltd, Ningbo, China) and a further i.p. injection 48 h later of 5 IU human chorionic gonadotropin (hCG, Ningbo Hormone Product Co., Ltd) after 48 h. For IVF, 12 h after hCG injection, mature oocytes were collected from the ampullae at 14 h post-hCG treatment, and *in vitro* fertilized by spermatozoa separated from ICR male epididymis. Then, zygotes were washed and cultured to the blastocyst stage in potassium simplex optimization medium containing amino acids (KSOM+AA, Millipore, Darmstadt, Germany) at 37 °C in 5% CO2 under 20% atmospheric oxygen concentration. To obtain IVO embryos, superovulated ICR female mice were cocaged individually with ICR males after the hCG injection.

The collection of IVO and IVF embryos was performed based on the well-established procedures and methods of our own published studies 13, 29-31. For preimplantation embryo collection, IVO embryos at the 8-cell, and morula stages (68-70, and 78-80 h after hCG, respectively) were recovered from donors by flushing the oviduct with M2 medium (formulation is shown in Table S7). The IVF blastocysts were harvested at 106-112 h post-hCG after culturing in KSOM medium, while control IVO blastocysts were harvested at 96-100 h post-hCG. In each group, well-developed embryos of similar morphology were selected for further analysis or embryo transfer.

For treatment with FGF2 or MEK/ERK inhibitor during *in vitro* culture, exogenous FGF2 (R&D, Minneapolis, MN, USA) or 5 μM MEK/ERK inhibitor PD0325901 (PD, Selleck, Houston, TX, USA) were supplemented into *in vitro* culture medium from the 4-cell stage, and embryos were collected at blastocyst stage for further analyses.

For postimplantation embryo collection, pseudo-pregnant ICR mice were cocaged individually with vasectomized with males 3.5 d before embryo transfer. Well-developed blastocysts with similar morphologies were selected for embryo transfer. Twelve blastocysts were transferred to each recipient, with six embryos in each uterine horn. E7.5 or E10.5 decidua were separated from uterus and fetuses, and embryonic parts of the E7.5 and E10.5 embryos were separated and washed by PBS before further analyses.

### High-throughput RNA sequencing (RNA-seq) and bioinformatic analysis

RNA-seq and bioinformatic analysis were performed based on the processes used in our previously published studies 31, 32. In the present study, 38394, 795 and 278 embryos in the IVF groups and 37271, 822 and 324 embryos in the IVO groups were sampled at E3.5, E7.5 and E10.5, respectively. Embryos at different stages were washed by ice-cold PBS for twice and cryopreserved into ultra-low temperature freezer till use. Total RNAs were extracted with TRIzol Reagent (Invitrogen, Carlsbad, CA, USA). Polyadenylated RNAs were isolated using the Oligotex mRNA Midi Kit (Qiagen, Valencia, CA, USA). The RNA-seq libraries were constructed using the SOLiD Whole Transcriptome Analysis Kit following the standard protocol (AB, Applied Biosystems, USA) and sequenced on the Applied Biosystems SOLiD platform to generate high-quality single-end reads. The raw reads were aligned to genome sequences, trimming off a nucleotide each from the 5' and 3' ends and allowing up to two mismatches. Reads mapped to multiple locations were discarded and only uniquely mapped reads were used for the subsequent analysis 13, 31, 32. Briefly, 73,107,951, 44,038,064 and 41,536,756 uniquely mapped reads in IVO embryos at E3.5, E7.5 and E10.5 were obtained with 39.83%, 46.88% and 47.07% mapping rate. Furthermore, 84,623,384, 40,616,266 and 45,876,283 uniquely mapped reads in IVF embryos at E3.5, E7.5 and E10.5 were obtained with 46.14%, 47.25% and 48.94% mapping rate. Gene expression levels were measured in reads per kilobase of exon model per million mapped reads (RPKM). Of note, due to the small amount of nucleic acid in each early embryo, we used a single pooling strategy to perform RNA-seq, thus, results in the present study did not have error bars 33, 34.

The Database for Annotation, Visualization and Integrated Discovery (DAVID v6.7; http://david.abcc.ncifcrf.gov) was used to annotate biological themes (Gene ontology, GO). The Kyoto Encyclopedia of Genes and Genomes (KEGG; http://www.genome.jp/kegg/) was used to determine the associated pathways. Phenotype annotations of differentially expressed genes were analyzed based on the Mouse Genome Informatics (MGI; http://www.informatics.jax.org/phenotypes.shtml) database.

### Methylated DNA immunoprecipitation-sequencing (MeDIP-seq)

In the present study, the same embryos were used to perform both RNA-seq and MeDIP-seq, which were demonstrated in our previous studies 32, 35. Briefly, Embryo samples were rinsed by PBS for twice and cryopreserved into ultra-low temperature freezer till use. Global genomic DNA, including both nuclear DNA (nDNA) and mitochondrial DNA (mtDNA), was isolated from embryo samples using Qiagen DNeasy kits (Qiagen), according to the modified manufacturer's instructions. The quality of each DNA sample was analyzed for integrity, purity and concentration on a Nanodrop Spectrophotometer DN-1000 (Nanodrop Technologies, Wilmington, DE, USA). Genomic DNA was then fragmented using a Covarias sonication system (Covarias, Woburn, MA, USA). After sonication, the fragments were denatured to produce single-stranded DNA (ssDNA). Following denaturation, the ssDNA was incubated with monoclonal 5-methyl-cytidine (5-mC) antibodies (Bio-rad, Minneapolis, MN, USA) at room temperature for 1 h. Magnetic beads conjugated to anti-mouse-IgG were then used to bind the anti-5-mC antibodies, and unbound DNA was removed along with the supernatant. Finally, 10 mg/ml proteinase K was used to digest the antibodies (60 °C 45 min, 95 °C 15 min), and the DNA was released and collected. Sequencing was carried out on the Illumina Hiseq 2000 (Illumina, San Diego, CA, USA) following the standard protocol to generate paired-end 50 bp reads by a commercial company (BGI, Shenzhen, Guangdong, China) 32, 35. Briefly, 84,075,585 and 86,570,768 uniquely mapped reads were obtained in IVO and IVF embryos at E7.5 with 51.50% and 53.02% mapping rates. Furthermore, 83,183,633 and 81,718,963 uniquely mapped reads were obtained in IVO and IVF embryos at E10.5 with 50.95% and 50.05% mapping rates. DNA methylation were measured in RPKM.

### Mouse ES cell culture and differentiation

Mouse ESCs line PGK12.1 was used in the present study. Cells were cultured in stem cell medium (SCM) containing Knockout DMEM base medium (Invitrogen), 10% fetal bovine serum (Hyclone, Logan, UT, USA), 1×non-essential amino acids (Millipore), 2 mM GlutaMAX (Invitrogen), 55 μM β-mercaptoethanol (Invitrogen), 1×10^5^ units leukemia inhibitory factor (LIF, Invitrogen) and 1% penicillin-streptomycin (Invitrogen), and incubated in a humidifier containing 5% CO_2_ at 37 °C. To induce differentiation, 3.0~5.0×10^3^ / cm^2^ cells were planted onto glasses coated by 5 μg/cm^2^ fibronectin in SCM. After 24 h growth, cells were rinsed by 1 ml PBS and differentiation medium, same as SCM without LIF, was added into each well to induce cell differentiation.

### Quantitative real-time polymerase chain reaction (qRT-PCR)

Embryos or cells used for RNA extraction were washed by PBS for twice and transferred into 1.5 ml centrifuge tube, and then, 1 ml TRIzol reagent were added. After 10 min incubation on ice, samples were cryopreserved into ultra-low temperature freezer till use. Total RNA was extracted from embryos or cells following the manufacturer's instructions with TRIzol reagent (Invitrogen). HiScript II Q Select RT SuperMix for qPCR kit (Vazyme, Nanjing, China) was used to reverse-transcribe 1 μg total RNA into cDNA according to the manufacturer's protocol. Quantitative real-time PCR analysis was performed using SsoFast EvaGreen Supermix (Bio-rad). Primers used in present study were exhibited in Table S1. Gene relative expression was referenced to *Gapdh* and calculated by the 2^-ΔΔCt^ method.

### Western blot

Embryos and cells used to detect protein level were rinsed by PBS for twice, and collected into centrifuge tube on ice, and 30 μl 2×lammli sample buffer (Bio-rad) with 5% β-mercaptoethanol was added following 100 °C boiled for 10 min. For differentiated cells, ice-cold PBS buffer was used to wash cells for three times, then RIPA buffer containing 1× protease cocktail (Beyotime Biotech. Inc., Shanghai, China) was added and cells were suspended and shake for several times on ice. Lysis solution was then centrifuged at 12500 ×g for 10 min and total concentration of supernatant protein was measure by BCA protein quantification kit (Beyotime Biotech. Inc.). 12% sodium dodecyl sulfate polyacrylamide gel electrophoresis (SDS-PAGE) was used to separate total protein samples and transferred to a PVDF membrane (Millipore) following 5% skim milk blocking for 1 h at room temperature. Primary antibody DNMT3B (1:200; GeneTex, Irvine, CA, USA) and β-tubulin (1:1000, Proteintech, Chicago, IL, USA) were diluted by blocking solution and the membranes were incubated overnight at 4 °C. Then, membranes were rinsed by TBST buffer for three times, and incubated with secondary antibodies conjugated with HRP for 1 h at room temperature. After three washes with TBST buffer, target protein bands were visualized using eECL Western Blot Kit (CWbio., Beijing, China) and detected by 5200 Imaging system (Tanon, Shanghai, China).

### Immunofluorescence analysis

4% paraformaldehyde in PBS was used to fix embryos (overnight at 4 °C) or cells (20 min at room temperature) after culture medium removing. After three washes by PBS, samples were permeabilizing with 0.5% Triton-X 100 for 0.5~1 h at room temperature, and blocking with 1% BSA-PBS at 4 °C for 2~6 h. For 5-mC staining, before blocking, permeabilized embryos or cells should be treated with 4 M Hcl for 20 min, and then neutralized with 100 Mm Tris-Hcl (pH = 8.0) for 30 min. Next, diluted primary antibodies DNMT3B (1:200, GeneTex), CDX2 (1:200; BioGenex, Fremont, CA, USA), NANOG (1:1000, Abcam, Cambridge, MA, USA) or 5-mC (1:250, Bio-rad) was used to incubate with samples overnight at 4 °C. After three washes by PBS, labeled secondary antibodies Alexa Fluor-488 (1:1000, Invitrogen) or Alexa Fluor-594 (1:1000, Invitrogen) was respectively added into samples under dark environment for 1 h at room temperature. Samples were then counterstained with DAPI, and imaged using an BX51 microscope (Olympus, Tokyo, Japan) accompanied with digital microscope camera (Olympus). All photographs were quantitated by Image J software (Rawak Software Inc., Stuttgart, Germany) and background subtraction was performed following the previous report 36.

### Chromatin immunoprecipitation (ChIP) assay

Differentiated cells were cross-linked with 1% formaldehyde and subjected to ChIP assay according to the protocols of ChromaFlash High-sensitivity ChIP kit (Epigentek, Farmingdale, NY, USA). The DNA lysate was crushed by ultrasonic breaker (Covarias) to produce 100~700 bps fragments. To obtain input controls, 1 μg DNA in each group was released and purified, and diluted to 10 times before use. Primary antibody SP1 (Santa Cruz Biotech., Santa Cruz, CA, USA) and negative control non-immune IgG were used to precipitate binding DNA fragments. Quantitative PCR was performed and enrichment was calculated as follows: % Input = 100 × 2^(Ct (adjusted input) - Ct (IP))^, Ct (adjusted input) = input Ct - 3.32. ChIP-qPCR primers were showed in Table S1.

### *Fgf4* gene knockout

CRISPR/CAS9n was used to knockout *Fgf4* gene in PGK12.1. Using online guide designer Benchling, we acquired and synthesized small guide RNA (sgRNA). sgRNAs were constructed to pSpCas9n(BB)-2A-Puro vector (PX462, purchased from Addgene) and transfected into PGK12.1 using Lipofectamine 2000 (Invitrogen) following the manufacturer's instructions. 24 h after transfection, 2 μg/ml puromycin was added into SCM to screen positive clones. Single clones were picked out after 5 days consistently screening, and PCR product sequencing was performed to identify knockout cell lines. All sgRNAs and PCR primers used for identification of knockout cell lines were showed in Table S1.

### Proliferation analysis

EdU staining was used to measure the proliferation of embryos in the present study. Following the protocols of BeyoClick™ EdU-594 Cell Proliferation Kit (Beyotime Biotech. Inc.), 10 μM EdU was added to KSOM medium and embryos were incubated at 37 °C for 2 h. After fixation and permeabilization, embryos were incubated with the Click Reaction mixture for 2 min and stained with DAPI. BX51 microscope (Olympus) and Image J software (Rawak Software Inc.) were respectively used to photograph and quantitate EdU intensity in each group.

### Statistical analysis

All experiments were replicated at least three times. Results were represented as means ± Standard Error of Mean (SEM) and analyzed with ANOVA, the Student's test or correct χ^2^ using the SPSS version 18.0 software (IBM Corp., Armonk, NY, USA). The *p*-value below 0.05 was considered as the threshold of significant statistics.

## Results

### IVF embryos exhibited global DNA hypomethylation by postimplantation stage

**Figure 1:**
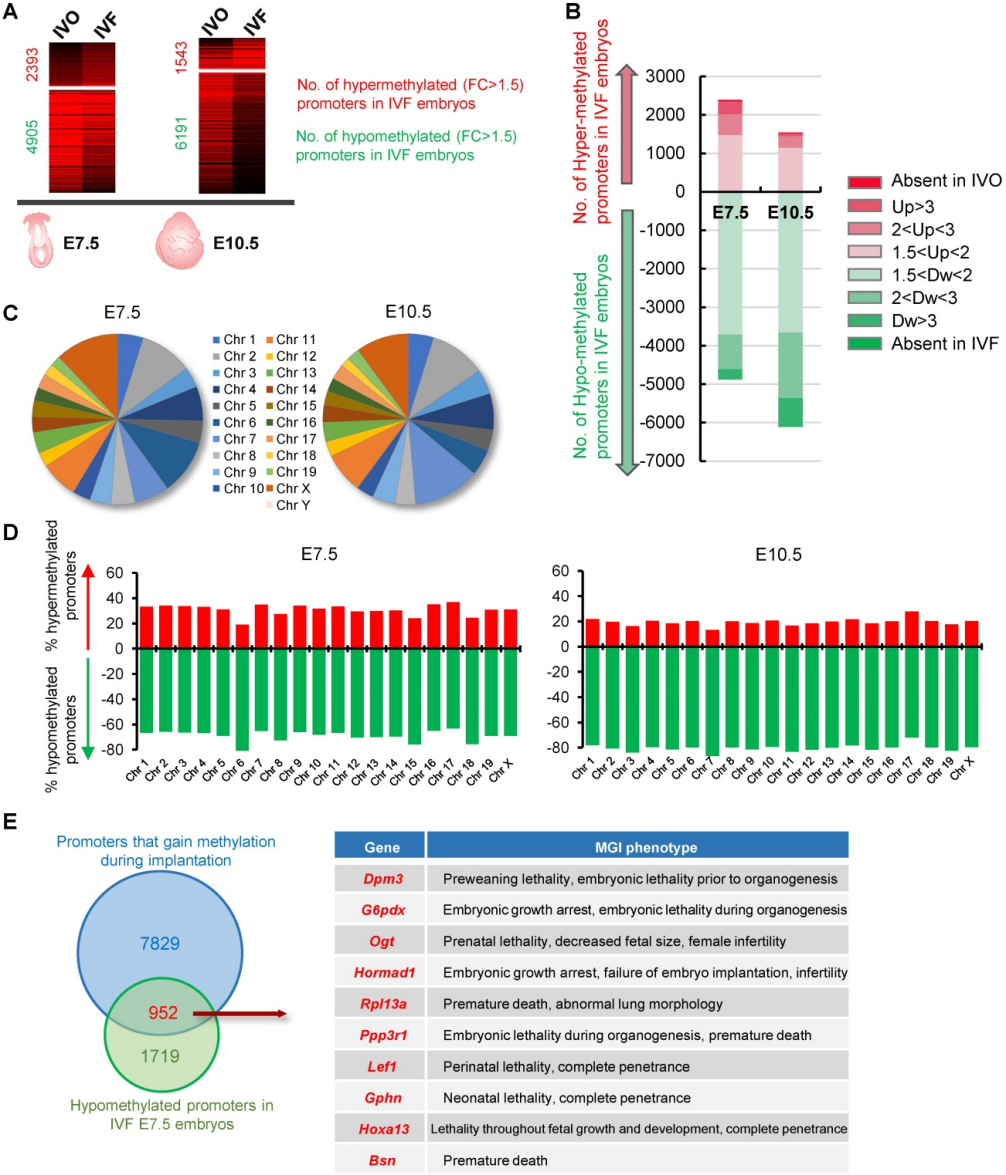
** IVF embryos exhibit global hypomethylation by postimplantation stage.** (A) Comparations of promoter DNA methylation in IVF or IVO embryos at E7.5 or E10.5. Red number and green numbers indicate hypermethylated (FC > 1.5) or hypomethylated (FC > 1.5) promoters in IVF embryos, respectively. Total data are shown in Table S5 and S6. 822 IVO and 795 IVF embryos at E7.5, 324 IVO and 278 IVF embryos at E10.5 were respectively used in this experiment. (B) Contribution of promoters with different fold changes in IVF embryos. (C) The chromosome-wide distribution of hypomethylated promoters in IVF embryos at E7.5 and E10.5 respectively. (D) Proportions of hypermethylated and hypomethylated promoters on each chromosome. (E) The Venn diagram of genes that should gain promoter methylation during implantation stage (FC E7.5/E3.5 > 1.5) and hypomethylated promoters in IVF E7.5 embryos (FC E7.5 IVF/IVO < 0.67). MGI phenotypes of overlapped genes are showed in right panel (top 10) and Table S2.

To test the effect of IVF process on DNA methylation patterns of postimplantation embryos, we compared our previously published global MeDIP-seq data of IVO and IVF embryos at E7.5 and E10.5 31, 32, 35, which have established relatively static DNA methylation patterns through the process of *de novo* DNA methylation 26, 27. We focused our analysis on promoter DNA methylation since promoters are main targets for transcriptional regulation of developmental genes in determining pluripotent or differentiated states 37, 38. 822 IVO and 795 IVF embryos at E7.5, 324 IVO and 278 IVF embryos at E10.5 were respectively used to perform MeDIP-seq. Compared with that in IVO embryos, a higher proportion of promoters were hypomethylated in IVF postimplantation embryos (Fig. 1A). Among the hypomethylated promoters in IVF embryos, a substantial proportion showed moderate (2 < FC < 3, 18.41% at E7.5 and 27.56% at E10.5) or dramatic (FC > 3 or absent in IVF, 5.93% at E7.5 and 13.31% at E10.5) decrease (Fig. 1B). Next, we detected the chromosome-wide distribution of hypomethylated promoter in IVF embryos at E7.5 and E10.5 respectively. Our results indicated that hypomethylated promoters were globally distributed across all autosomes and sex chromosomes (Fig. 1C), and the number of hypomethylated promoters was considerably higher than that of hypermethylated promoters on each chromosome (Fig. 1D). Next, a Venn diagram showed that a substantial proportion of promoters that should gain DNA methylation (upregulated from E3.5 to E7.5 with FC > 1.5) during implantation were hypomethylated in IVF E7.5 embryos (Fig. 1E). Phenotype annotations from Mouse Genome Informatics (MGI) database further demonstrated that majority of those genes were functionally involved in embryonic developmental and survival, as well as postnatal growth. Collectively, these results indicate that IVF embryos may undergo impaired *de novo* DNA methylation during implantation, and thus leading to global hypomethylation by postimplantation stage.

### Impaired *de novo* DNA methylation transcriptionally participate in processes essential for embryonic development and survival

**Figure 2:**
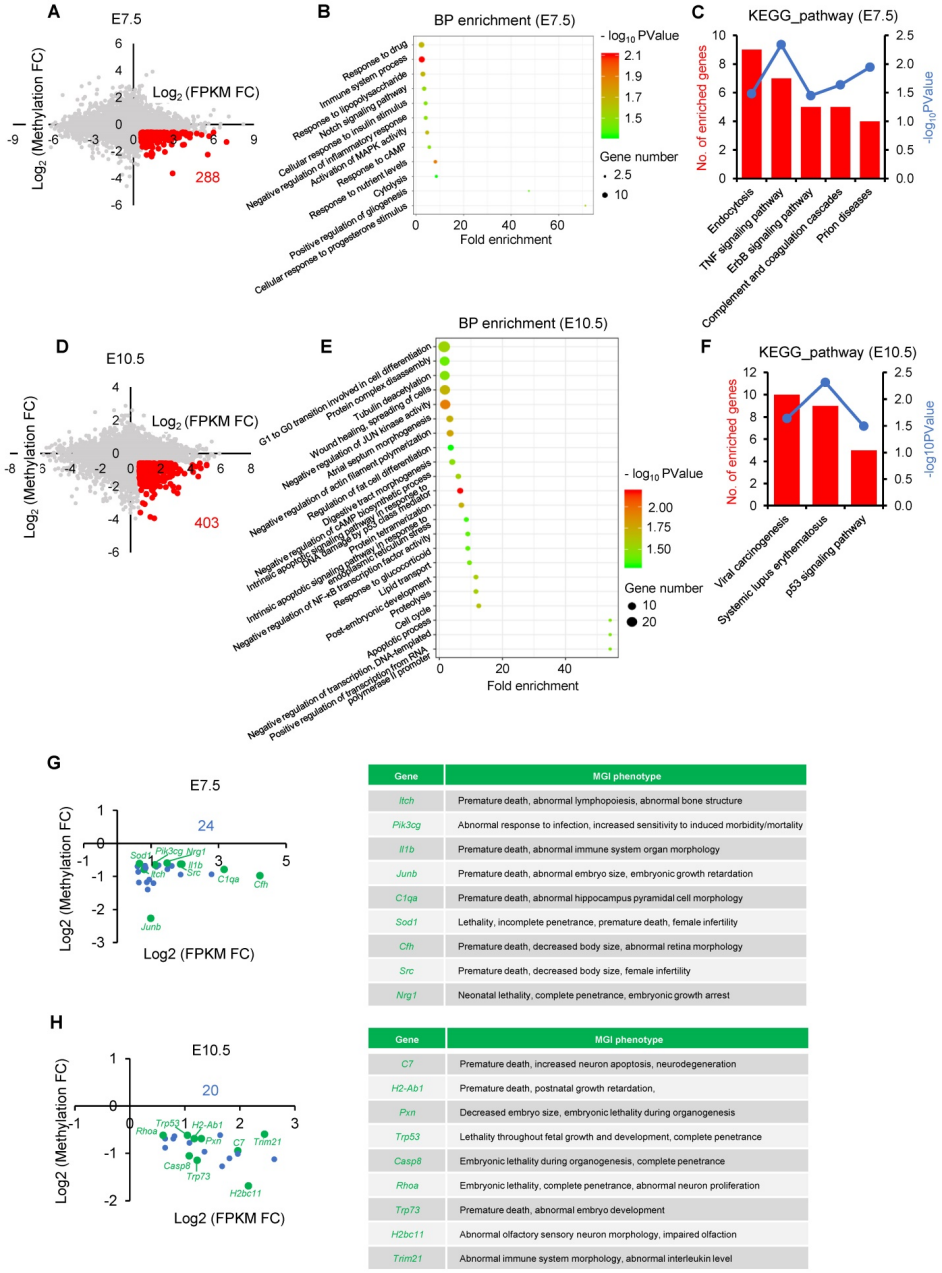
** Functional analysis of dysregulated genes due to hypomethylated promoters in IVF postimplantation embryos.** (A and D) A scatter plots of putative genes (red dots, Log_2_ FPKM FC > 0.58) that are dysregulated due to impaired *de novo* DNA methylation (Log_2_ Methylation FC < -0.58) in IVF embryos at E7.5(A) or E10.5 (D), respectively. (B and E) Biological process (BP) enrichment based on functional annotation of GO terms using filtered genes at E7.5 (B) or E10.5 (E). (C and F) KEGG analysis of genes enriched by BP enrichment at E7.5 (C) or E10.5 (F). The left ordinate indicates the number of enriched genes in each term, and the right ordinate indicates enrichment sore. (G and H) The location of genes enriched by KEGG analysis at E7.5 (G) or E10.5 (H). Genes that have MGI phenotypes related to embryos development are highlighted as green dots. Complete MGI phenotypes are showed in Table S3 and Table S4.

Given DNA methylation at postimplantation stage plays an essential role in transcriptional regulation of developmental genes 26, 39, we next attempted to test the developmental consequence of impaired *de novo* DNA methylation. To this end, we performed an integrated analysis using our previously published MeDIP-seq data and transcriptome data that profiles DNA methylation and gene expression maps of IVO and IVF postimplantation embryos, respectively 31, 35. There were 288 and 403 putative genes that were dysregulated due to impaired *de novo* DNA methylation at E7.5 and E10.5 respectively (Fig. 2A and D). GO and KEGG analyses indicated that those genes participated in many basic processes and pathways, such as endocytosis, cytolysis, cell differentiation, Notch and MAPK signaling pathways, as well as cellular response to stress stimulus (Fig. 2B, C, E, F). Moreover, based on MGI database, we found that genes enriched in KEGG analysis, were tightly associated with embryonic development and survival throughout the pregnancy (Fig. 2G and H).

### *Dnmt3b* is consistently inhibited during the transition from the blastocyst to postimplantation stage in IVF embryos

**Figure 3:**
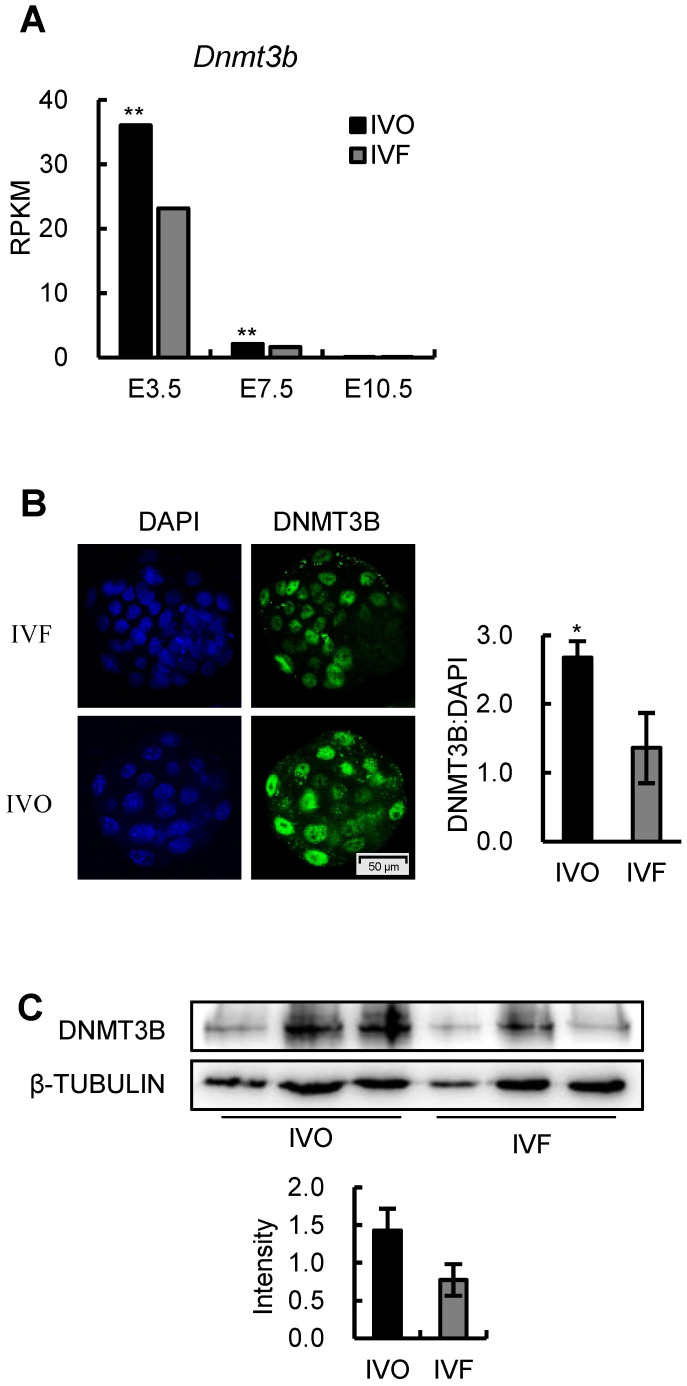
***Dnmt3b* is consistently inhibited during the transition from the blastocyst to postimplantation stage in IVF embryos.** (A) Comparison of expression levels of *Dnmt3b* between IVO or IVF embryos at E3.5, E7.5 or E10.5 using transcriptome data. (B) Immunofluorescence staining of DNMT3B in IVO (24 embryos) or IVF (21 embryos) blastocysts and relative quantification of DNMT3B signals (right panel). (C) Western blot analysis of DNMT3B in IVO or IVF E7.5 embryos and relative quantification of DNMT3B levels (lower panel). Three pools were used to perform WB in IVO or IVF group, with 3 embryos in each pool. *P < 0.05, **P < 0.01.

Given the DNMT3B is the main enzyme required for *de novo* DNA methylation during early development 26, 28, we next attempted to test if *Dnmt3b* was inhibited in IVF embryos during early development. Analysis of dynamic transcriptome of IVO and IVF embryos revealed that *Dnmt3b* was consistently inhibited during the transition from the blastocyst to postimplantation stage (Fig. 3A). In addition, the inhibited *Dnmt3b* expression was further confirmed on the protein level in the blastocyst (Fig. 3B) and E7.5 embryos (Fig. 3C). Those results demonstrate that IVF processes induce a significant decrease in *Dnmt3b*/DNMT3B in early embryos, which may be the potential target for correcting DNA methylation defects in IVF postimplantation embryos.

### Repression of FGF signaling is responsible for inhibited *Dnmt3b* expression in IVF preimplantation embryos

**Figure 4:**
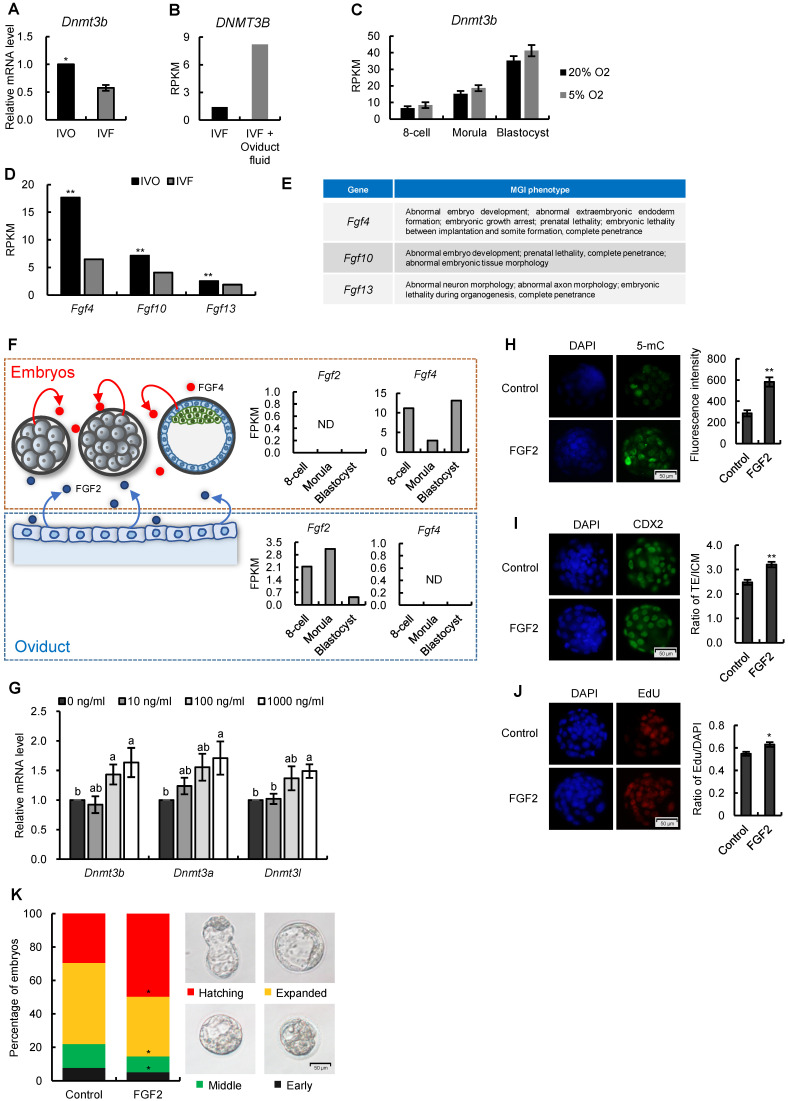
** Repression of FGF signaling is responsible for inhibited *Dnmt3b* expression in IVF preimplantation embryos.** (A) Relative mRNA expression levels of *Dnmt3b* in IVO and IVF blastocysts using RT-qPCR analysis. Experiments were replicated three times. At least 25 embryos were collected in each group. (B) Comparison of *DNMT3B* expression levels in IVF and IVF + oviduct fluid porcine embryos using previously published transcriptome data 40. (C) Dynamic comparisons of *Dnmt3b* expression levels using transcriptome data of IVF mouse preimplantation embryos under 20% or 5% O_2_ condition. (D) Comparison of *Fgf4*, *Fgf10* and *Fgf13* expression levels in IVO and IVF blastocysts. (E) MGI phenotypes of main FGF members that are inhibited in IVF blastocysts. (F) Diagram of expression patterns of autocrine and paracrine FGF ligands during preimplantation stage. Right panel in each box shows expression levels of autocrine and paracrine FGF ligands in embryos themselves and the oviducts. (G) Expression levels of *Dnmt3b*, *Dnmt3a* and *Dnmt3l* in IVF blastocysts cultured in medium supplemented with 10 ng/ml, 100 ng/ml or 1000 ng/ml FGF2. Experiments were replicated four times. 30~56 embryos were collected in each group. (H) Immunofluorescence staining of 5-mC in IVF blastocysts cultured in control medium (33 embryos) or medium supplemented with 1000 ng/ml FGF2 (31 embryos). Right panel shows relative fluorescence intensity of 5-mC (5-mC/DAPI). (I) Immunofluorescence staining of CDX2 in IVF blastocysts treated with (44 embryos) or without (6 embryos) exogenous FGF2. Right panel shows ratio of TE/ICM in IVF blastocysts treated with or without exogenous FGF2. (J) EdU staining (left) and quantification of the ratio of EdU/DAPI (right) in IVF blastocysts treated with (39 embryos) or without (39 embryos) exogenous FGF2. (K) Percentage of embryos at different developmental stages in the culture medium with or without exogenous FGF2. Experiments were replicated five times. 39~69 embryos were collected in each group. Representative embryo pictures were showed in the right panel. Values with different letters are significantly different (*P* < 0.05). *P < 0.05, **P < 0.01.

Having confirmed the consistent repression of *Dnmt3b*/DNMT3B in early IVF embryos, we next attempted to identify the critical reasons that contributed to *Dnmt3b* inhibition in IVF embryos. Given the transcriptome data suggested *Dnmt3b* was transcriptionally inhibited as early as the preimplantation stage (Fig. 3A) we first confirmed this inhibition in IVF blastocysts using RT-qPCR analysis (Fig. 4A). In addition, by reanalyzing previously published 40 and our own transcriptome data, we found medium components (Fig. 4B), but not oxygen concentration of culture conditions (Fig. 4C), can increase *Dnmt3b* expression.

Since previous studies demonstrated that FGF signaling plays a critical role in stimulating *Dnmt3b* expression 41-43, we next profiled expression patterns of FGF family members in early embryos and maternal oviduct, using our transcriptome data. We found many FGF ligands that are tightly associated with embryonic development and survival was repressed in IVF embryos (Fig. 4D and E). In addition, we noticed that FGF2, which can substitute for FGF4 to rescue the developmental defects in FGF4-deficient mouse embryos 44, was exclusively produced maternal oviduct (Fig. 4F). This observation, together with fact that oviduct fluid supplementation can stimulate *Dnmt3b* expression (Fig. 4B), led us to ask if FGF2 supplementation can rescue *Dnmt3b* inhibition in IVF embryos. To test this, standard *in vitro* culture medium was supplemented with FGF2 at different concentrations. As expected, FGF2 supplementation significantly increased the expression levels of *Dnmt3b*, as well as *Dnmt3a* and *Dnmt3l* (Fig. 4G), and thus enhanced DNA methylation levels in IVF embryos (Fig. 4H). In addition, immunofluorescence analysis of CDX2 and EdU staining indicated that FGF2 also facilitate trophectoderm (TE) differentiation (Fig. 4I) and proliferation (Fig. 4J) during blastocyst formation. Finally, morphological quantifications also showed the beneficial effect of FGF2 supplementation in accelerating developmental progression in IVF embryos (Fig. 4K). Collectively, these results suggest that repression of FGF signaling, especially the deficiency of paracrine FGF2 from oviduct during preimplantation stage, may be the main cause of *Dnmt3b* inhibition in IVF blastocysts.

### FGF signaling remains repressed in IVF postimplantation embryos and induces DNA hypomethylation during early development

**Figure 5:**
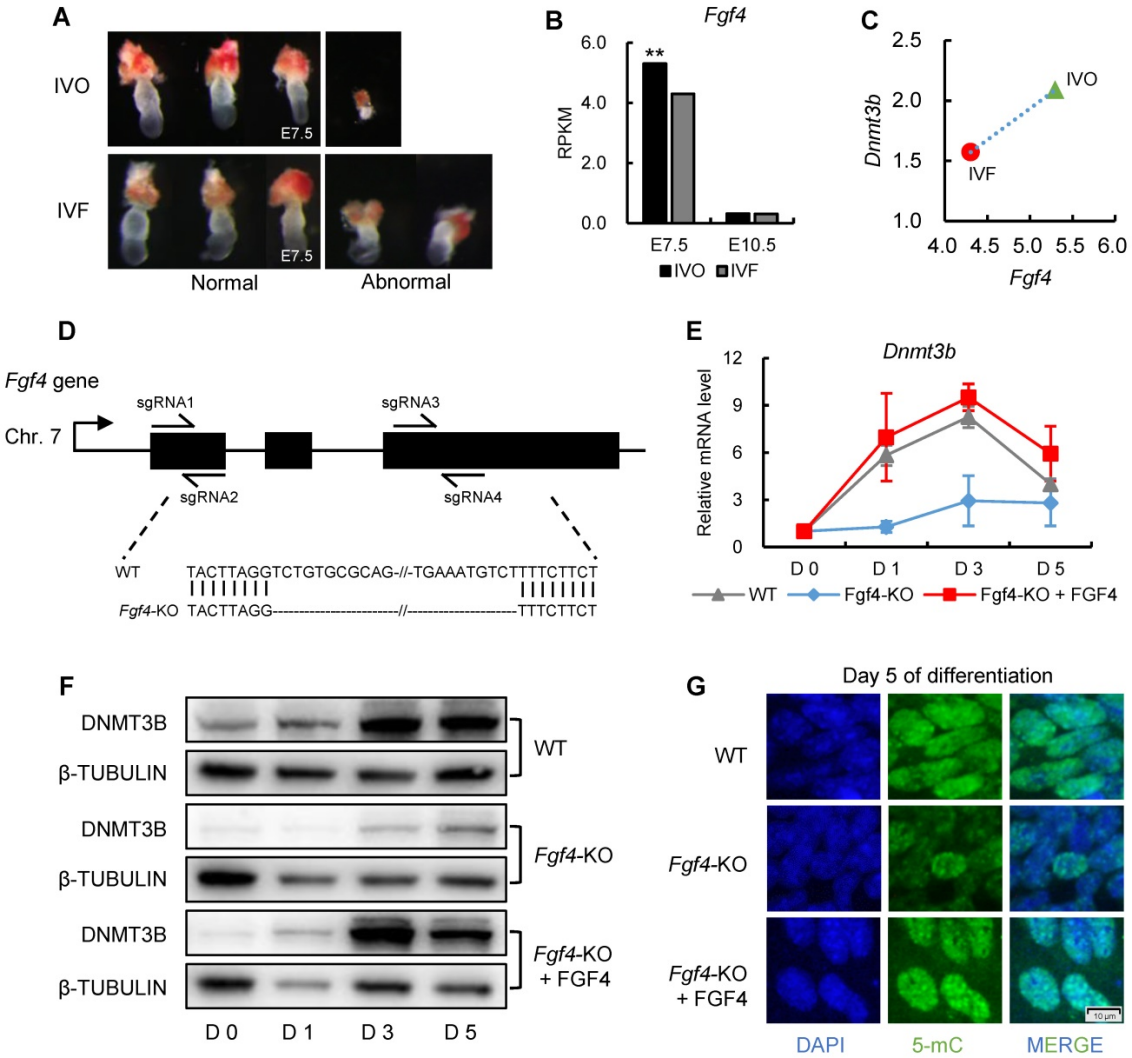
** FGF signaling remains repressed in IVF postimplantation embryos and induces DNA hypomethylation during early development.** (A) Representative images of morphologically normal and abnormal IVO or IVF embryos at E7.5. (B) Comparison of expression levels of *Fgf4* between IVO or IVF embryos at E7.5 or E10.5. (C) The positive correlation between *Fgf4* and *Dnmt3b* expression levels in IVO and IVF E7.5 embryos. (D) Flow diagram of CRISPR/CAS9n-based knockout of *Fgf4*. (E and F) The relative mRNA (E) or protein (F) levels of *Dnmt3b*/DNMT3B in WT, *Fgf4*-KO, or *Fgf4*-KO+FGF4 ES cells on Day 0, 1, 3 and 5 of differentiation. Experiments were triple replicated, and representative WB images were exhibited. (G) Immunofluorescence staining of 5-mC in WT, *Fgf4*-KO or *Fgf4*-KO + FGF4 on Day 5 of differentiation. ** *P* < 0.01.

Having established a correlation between repression of FGF signaling and *Dnmt3b* inhibition in preimplantation embryos, we next attempted to address whether FGF signaling remains repressed in IVF embryos following transfer to recipients. The remarkable morphological abnormalities of IVF embryos were observed as early as the stage shortly after implantation (Fig. 5A), which is similar with that reported our previous study 13. Given that FGF4 is a key factor required for embryonic development following blastocyst stage and essential for successful implantation 44-46, we next compared *Fgf4* expression between IVO and IVF postimplantation embryos using our transcriptome data. We found *Fgf4* remained repressed in IVF E7.5 embryos (Fig. 5B), which was positively correlated with the *Dnmt3b* inhibition at this stage (Fig. 5C).

Next, we attempted to determine if consistent repression of FGF signaling is responsible for *Dnmt3b* inhibition and DNA hypomethylation in IVF postimplantation stage. Because *in vivo* intervention of embryonic FGF signaling is technically inefficient, we used mouse ES cell differentiation as a model, which recapitulated the process of *de novo* DNA methylation during implantation 47, 48. It should be mentioned that ES cell differentiation was induced in serum-free medium, to exclude the possible influence of serum FGF ligand. CRISPR/CAS9n-based genome editing system was used to knockout *Fgf4* gene in mESCs (Fig. 5D). Comparing with wild type cells, the expression levels of *Dnmt3b* gene in *Fgf4*-knockout (*Fgf4*-KO) ES cells were consistently inhibited during differentiation, which can be completely restored by exogenous supplementation of FGF4 (Fig. 5E). These findings were also confirmed on protein levels (Fig. 5F). Correspondingly, results of 5-mC staining of 5 days differentiated *Fgf4*-KO ES cells showed a remarkable DNA hypomethylation (Fig. 5G), suggesting that repression of FGF signaling impairs the process of *de novo* DNA methylation.

### FGF signaling regulates *Dnmt3b* expression in early embryos through MEK/ERK-SP1 pathway

**Figure 6:**
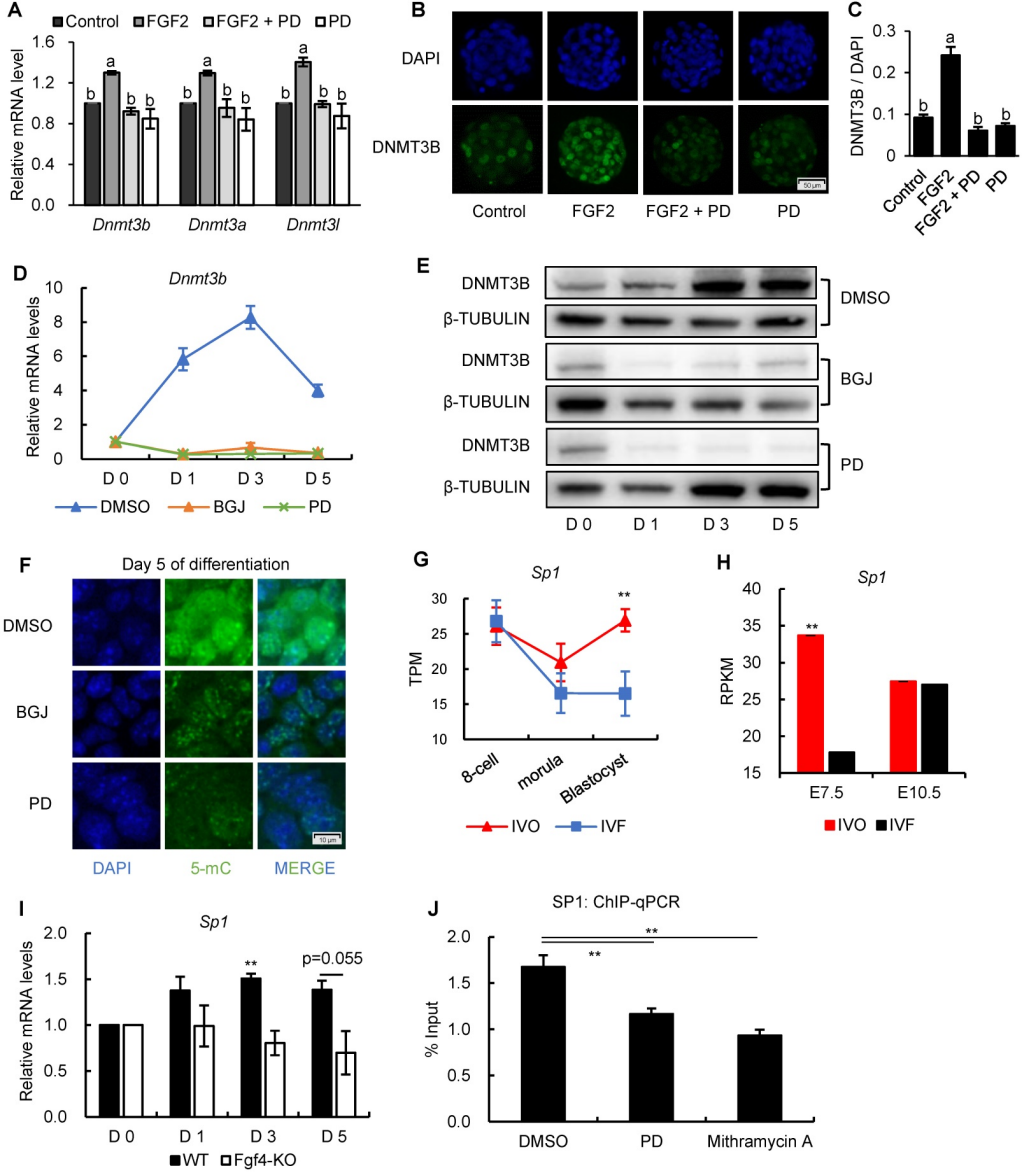
** FGF signaling regulates *Dnmt3b* expression early development through MEK/ERK-SP1 pathway.** (A) Relative expression levels of *Dnmt3b*, *Dnmt3a* and *Dnmt3l* in IVF blastocysts treated with FGF2, FGF2+PD (MEK/ERK inhibitor) and PD respectively. Experiments were replicated three times. 18~43 embryos were collected in each group. (B and C) Immunofluorescence staining (B) and quantification (C) of DNMT3B in IVF blastocysts treated with FGF2 (42 embryos), FGF2+PD (30 embryos) and PD (30 embryos) respectively. 63 embryos were used as control. (D and E) Relative mRNA (D) and protein (E) levels of *Dnmt3b*/DNMT3B in ES cells treated with DMSO, BGJ (nonspecific FGFR inhibitor) and PD on Day 0, 1, 3 and 5 of differentiation. Experiments were triple replicated, and representative WB images were exhibited. (F) Immunofluorescence staining of 5-mC in ES cells treated with DMSO, BGJ and PD on Day 5 of differentiation. (G and H) Dynamic comparisons of expression levels of *Sp1* in IVO or IVF embryos during preimplantation (G) and postimplantation (H) stage using transcriptome data. (I) Dynamic comparisons of expression levels of *Sp1* in WT or *Fgf4*-KO ES cells on Day 0, 1, 3 and 5 of differentiation. Experiments were triple replicated. (J) ChIP analysis of SP1 enrichment at a distal binding site that is located 10 kb upstream of *Dnmt3b* in ES cells treated with DMSO, PD and Mithramycin A (nonspecific SP1 inhibitor). ChIP-qPCR were repeated for three times. Values with different letters are significantly different (P < 0.05). *P < 0.05, **P < 0.01.

To further understand the mechanism underlying *Dnmt3b* inhibition and impaired *de novo* DNA methylation which could be used as the potential target for correcting developmental defects of IVF embryos, we next tested the involvement of ERK signaling, the primary downstream pathway of FGF signaling, in mediating FGF-induced *Dnmt3b* expression. The beneficial effect of FGF2 supplementation on increasing *Dnmt3b* expression in IVF blastocyst, could be completely attenuated by supplementing MEK/ERK inhibitor PD0325901 (PD) (Fig. 6A). This result was further confirmed on protein levels using immunofluorescence staining of DNMT3B (Fig. 6B and C). Correspondingly, both mRNA and protein analyses from ES cells showed that *Dnmt3b*/DNMT3B expression were completely absent when FGFR and MEK/ERK pathway were blocked by inhibitors respectively (Fig. 6D and E). In addition, we also found either FGFR or MEK/ERK inhibition led to DNA hypomethylation in 5 days differentiated ES cells (Fig. 6F). Those results demonstrated that FGF signaling regulates *Dnmt3b* expression and DNA methylation levels during early development through FGFR-MEK/ERK pathway.

Because results from previous study showed that a general transcription factor specificity protein 1 (SP1) participates in the transcriptional regulation *Dnm3b* 49, we next test the involvement of SP1 in *Dnmt3b* inhibition. Results from transcriptome data showed that *Sp1* was consistently inhibited in IVF embryos from preimplantation to postimplantation stage (Fig. 6G and H), which coincides with the FGF signaling and *Dnmt3b* inhibition. More importantly, using *Fgf4*-KO ES cells, we found transcriptional upregulation of *Sp1* was also largely dependent on FGF4 during ES differentiation (Fig. 6I), indicating that SP1 may play an important role in mediating FGF-MEK/ERK-induced *Dnmt3b* expression during early development. To provide more direct evidence supporting the mediating role of SP1 in regulating *Dnmt3b* expression, we screened *Dnmt3b* promoter regions and SP1-ChIP data in Cistrome Data Browser. Results revealed several potential SP1 binding sites in *Dnmt3b* promoter regions. Results from high-sensitivity immunoprecipitation showed that SP1 are significantly enriched in *Dnmt3b* promoter regions, but the binding could be significantly attenuated by blocking MEK/ERK pathway, or supplementing mithramycin A, a non-specific inhibitor that displaces transcriptional activators which bind to GC-rich regions of promoters such as SP1 (Fig. 6J). These results demonstrated that FGF signaling-induced *Dnmt3b* expression during early development is largely dependent on MEK/ERK-SP1 pathway.

## Discussion

It has been widely accepted that a wave of genome-wide* de novo* DNA methylation that occurs around implantation period, re-establishes the DNA methylation patterns from a globally hypomethylated state in blastocysts to relatively static DNA methylation patterns in postimplantation embryos 26, 27, 50. Focusing on promoters, which are main targets for transcriptional regulation of developmental genes in determining pluripotent or differentiated states 37, 38, and are preferentially* de novo* methylated during early development 26, 39, we find IVF embryos undergo an impaired *de novo* DNA methylation. According to previous studies using *Dnmts*-deficient mouse models, defects in *de novo* DNA methylation result in severe embryonic or postnatal lethality 28, 51, as well as aberrant organogenesis 52, 53. This is in line with the morphological observation in our previous studies 13, 31, as well as the integrated bioinformatic analysis in the present study: many putative genes that were dysregulated due to impaired *de novo* DNA methylation participated in many basic processes and pathways (*e.g.*, cell differentiation, cellular response to stress stimulus, Notch and MAPK signaling pathways, *etc*.) and were tightly associated with embryonic development and survival throughout the pregnancy.

DNA methyltransferase DNMT3B is the main enzyme required for *de novo* DNA methylation during early development. The genetic depletion of *Dnmt3b* leads to severe hypomethylation at pluripotency genes, gastrulation genes, germline-specific genes, hematopoietic genes, etc. 26, 39. In addition, *Dnmt3b*-deficient mice show multiple developmental defects shortly after implantation and postimplantation embryonic lethality 28, 54. In the present study, the observation of consistent *Dnmt3b* inhibition in IVF embryos, together with results of our own and other colleagues' studies 55, 56, suggest that the expression of methyltransferase is highly sensitive to perturbations of developmental environment. Correspondingly, we observed a global hypomethylation in IVF postimplantation embryos, indicating that IVF embryos undergo an impaired *de novo* DNA methylation during implantation stage. These results are in line with previous observations of genomic hypomethylation or global imprinting loss in IVF-conceived embryos, placentas, and offspring 17, 19, 57, since* Dnmt3b* was also thought to be involved in the maintenance of DNA methylation imprints during early development 28.

Our study further indicates that repression of FGF signaling is the main cause of *Dnmt3b* inhibition in IVF embryos during the transition from the blastocyst to postimplantation stage. Indeed, the critical mechanism responsible for initiation and maintenance of *Dnmt3b* expression during early development remains largely unknown. Although a previous study suggested that FGF signaling inhibition in ES cells induced repression of *de novo* methyltransferases, as well as global hypomethylation, via chemical-induced ERK1/2 and GSK3B signaling inhibition (2i) 41, the precise upstream signaling responsible for transcriptional activation of *Dnmt3b* remains elusive. Our study, using blastocysts and ES cells as models, indicates that FGF signaling is critical for initiation and maintenance of *Dnmt3b* expression. FGF signaling in the blastocysts is connection with the exit from pluripotency to differentiation. In particular, FGF4, which is the only FGF ligand produced by ICM cells in the blastocysts, is indispensable for lineage differentiation of pluripotent epiblast (Epi) and the primitive endoderm (PrE), the second fate decision of early embryos, and thus is required for postimplantation development 44, 46, 58, 59. The coincidence between this process and *de novo* DNA methylation raises the possibility that FGF signaling is also essential for stimulating *Dnmt3b* expression during early development. Evidences from *Fgf4*-deficient ES cells support this hypothesis: *Dnmt3b* expression fails to be imitated and remains at low levels throughout differentiation, and this failure can be completely rescued by exogenous supplementation of FGF4. Of note, MEK/ERK-SP1 pathway plays a critical mediating role in this process. Considering that activation of MEK/ERK signaling mediates the effect of growth factors and is essential for early development and differentiation 60-62, MEK/ERK-SP1 pathway may be a potential target for rescuing developmental defects of IVF embryos.

In addition to consistently inhibited *Fgf4* expression in IVF embryos during the transition from the blastocyst to postimplantation stage, we also noticed that the absence of paracrine FGF signaling may also participate in *Dnmt3b* inhibition. FGF2 is a representative paracrine factor that is exclusively produced by the oviduct, but not by embryos during early development. Our results indicate that supplementation of FGF2, which is not present in any commercially available culture media, can restore *Dnmt3b* expression to the level comparable to that of IVO embryos, and this beneficial effect can be blocked by inhibiting MEK/ERK pathway. It should also be mentioned that in addition to upregulated *Dnmt3b* expression and increased DNA methylation levels, FGF2 supplementation also changed lineage differentiation and proliferation of blastocysts. These observations, further emphasized the importance of DNA homeostasis in finetuning preimplantation development, as proposed by previous studies 41, 63, 64.

The beneficial effect of paracrine FGF2, are in agree with the results reported by P Coy *et al*: DNA methylation and gene expression of IVF embryos can be partially corrected in the presence of oviductal fluid 40, 65. Our results, together with results reported by P Coy *et al.*, support the concept that establishment and maintenance of epigenetic features of early embryos may be achieved via the synergic effect of autocrine and paracrine factors, which are produced by embryos themselves and the oviduct, respectively. Given safety concerns of transmission of diseases have not fully been addressed after addition of oviductal fluids in the culture media, it only been applied in animal reproduction at present, but not clinical use. By contrast, the chemically defined culture medium that enhances developmental potential of IVF embryos is more reasonable, especially in the context of clinical practice of human assisted reproductive technologies. Until now, however, only very limited growth factors or cytokines that present in oviductal fluid are proven to be used in commercially available culture media 66. Thus, identifying the developmental role of oviductal growth factors or cytokines in supporting early embryos, and thus formulating the culture media, may be a promising strategy.

**Figure 7:**
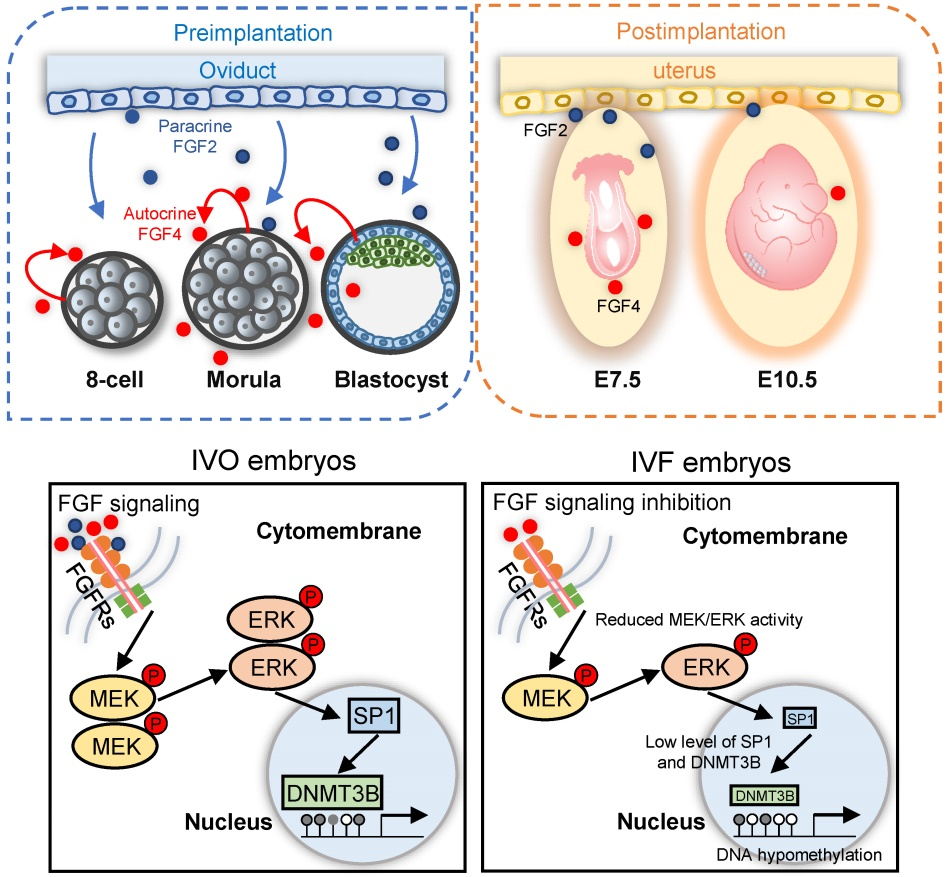
** Schematic diagram illustrating the role of repression of FGF signaling in inducing *Dnmt3b* inhibition during early development of IVF embryos.** Upper panels: early embryonic development during preimplantation stage are supported via the synergic effect of autocrine and paracrine FGF signaling. Blue and red dots represent FGF2 and FGF4 ligands, which are produced by the oviduct and embryos themselves, respectively. Lower panel: repression of FGF signaling underlies the mechanism responsible for *Dnmt3b* inhibition and hypomethylation in IVF embryos, MEK/ERK-SP1 pathway plays an essential mediating role during this process.

Collectively, focusing on DNA hypomethylation in IVF postimplantation embryos, our study identifies that FGF signaling is essential for supporting *Dnmt3b* expression during early development, via the synergic effect of autocrine and paracrine FGF ligands. During *in vitro* development, repression of FGF signaling is responsible for consistent inhibition of* Dnmt3b*, the main *de novo* methyltransferase (Fig. 7). Our current finding not only reveals the main mechanism underlying DNA methylation defects in IVF embryos, but also proposes a potential strategy for preventing or correcting IVF-associated epigenetic errors via the use of oviductal growth factors or cytokines that can support early embryonic development.

## Supplementary Material

Supplementary figures and tables.
